# Chromatin Architecture as an Essential Determinant of Dendritic Cell Function

**DOI:** 10.3389/fimmu.2019.01119

**Published:** 2019-06-04

**Authors:** Giselle M. Boukhaled, Mario Corrado, Hannah Guak, Connie M. Krawczyk

**Affiliations:** ^1^Department of Physiology, Goodman Cancer Research Center, McGill University, Montreal, QC, Canada; ^2^Center for Cancer and Cell Biology, Program in Metabolic and Nutritional Programming, Van Andel Institute, Grand Rapids, MI, United States

**Keywords:** dendritic cells, epigenetics (MeSH), metabolism, inflammation, tolerance, microenvironment

## Abstract

Epigenetics has widespread implications in a variety of cellular processes ranging from cell identity and specification, to cellular adaptation to environmental stimuli. While typically associated with heritable changes in gene expression, epigenetic mechanisms are now appreciated to regulate dynamic changes in gene expression—even in post-mitotic cells. Cells of the innate immune system, including dendritic cells (DC), rapidly integrate signals from their microenvironment and respond accordingly, undergoing massive changes in transcriptional programming. This dynamic transcriptional reprogramming relies on epigenetic changes mediated by numerous enzymes and their substrates. This review highlights our current understanding of epigenetic regulation of DC function. Epigenetic mechanisms contribute to the maintenance of the steady state and are important for precise responses to proinflammatory stimuli. Interdependence between epigenetic modifications and the delicate balance of metabolites present another layer of complexity. In addition, dynamic regulation of the expression of proteins that modify chromatin architecture in DCs significantly impacts DC function. Environmental factors, including inflammation, aging, chemicals, nutrients, and lipid mediators, are increasingly appreciated to affect the epigenome in DCs, and, in doing so, regulate host immunity. Our understanding of how epigenetic mechanisms regulate DC function is in its infancy, and it must be expanded in order to discern the mechanisms underlying the balance between health and disease states.

## Introduction

Epigenetics refers to the regulation of gene expression by mechanisms other than changes in DNA sequence. Epigenetic mechanisms enable long-term phenotypic responses to the environment in the absence of initiating stimuli. Historically, epigenetic memory has referred to stable changes that are maintained through cell division. However, it is increasingly appreciated that dynamic changes in the epigenome, including in the absence of cell division, are equally important for proper cellular function.

Dendritic cells (DCs) are phagocytic cells of the innate immune system that reside in nearly every tissue and specialize in antigen presentation. They are rapidly responsive to stimuli including infection, inflammation, cancer, particles and cellular damage, are highly migratory, and direct the nature of ensuing immune responses by producing context-specific factors such as cytokines. As for most cells, epigenetic mechanisms underpin lineage specification of DCs. There are several subsets of DCs, all of which are derived from a common DC progenitor (CDP). CDPs give rise to plasmacytoid DCs (pDCs) and pre-DCs, the latter of which differentiates into conventional DCs (cDC1s and cDC2s) in secondary lymphoid tissues (1–6). pDCs produce high levels of type 1 interferons (IFNs) during antiviral and anti-tumor responses. cDCs are highly-specialized antigen-presenting cells; cDC1s (XCR1^+^) specialize in antigen cross-presentation and stimulation of CD8^+^ T cells and Th1 responses, whereas cDC2s (CD11b^+^ CD172a^+^) specialize in antigen presentation to CD4^+^ T cells and direct responses to extracellular pathogens (7–10). Additionally, during active inflammation, monocytes can acquire the function of macrophages or DCs (moDCs) (8). The transcriptional mechanisms controlling lineage commitment and DC diversity have been extensively studied. Lineage-determining factors such as PU.1 and C/EBP are significant regulators of myeloid cell differentiation. They facilitate lineage specification of hematopoietic cells by forming stable interactions with their chromatin substrates, enabling secondary factors to drive lineage-specific gene expression (11–14). The complexity of lineage-determining factors and their roles in specifying DC fate through regulation of chromatin remodeling and gene expression have been described elsewhere and is not addressed here (15, 16).

DCs are relatively rare, and thus a number of *in vitro* culture systems have been developed to study their function (17). While the cells generated in these cultures do not perfectly reflect cells found *in vivo*, their experimental use has significantly advanced our knowledge of DC biology. Human DC cultures are typically monocyte-derived and generated by culturing blood monocytes with GM-CSF and IL-4 (18). For mouse, bone marrow can be cultured with combinations of GM-CSF with or without IL-4 to give rise to heterogeneous cultures of bone marrow-derived DCs (BMDCs) that possess cDC- and macrophage-like qualities (19–22). Culturing bone marrow with FLT3L gives rise to a mixed culture containing both cDC- and pDC-like cells (23–26). More recently, the addition of Notch-ligands to the *in vitro* culture system gives rise to cells that are more phenotypically similar to cDC1s and cDC2s (27). Because of the ease of generating BMDCs and the feasibility of generating large numbers of cells, BMDCs are frequently used for biochemical studies, including those addressing epigenetic and metabolic mechanisms.

Further to differentiation, dynamic epigenetic regulation is inherent to the massive transcriptional reprogramming required to orchestrate an effective and efficient immune response (28–31). In steady-state BMDCs, transcription factors (TFs), including ATF3, IRF4, and JUNB, were discovered to serve as priming factors for genes that are rapidly induced following TLR stimulation (11). Priming factors are present at accessible promoters and enhancers in the absence of stimulation. Upon stimulation, priming factors facilitate induced gene expression, possibly by serving as docking sites for dynamic factors or by maintaining chromatin accessibility of regulatory elements for other factors (11, 32). Epigenetic regulation of gene expression is also important for communicating context. Context is inferred by cell surface receptors such as pattern recognition receptors (PRRs) and cytokine/chemokine/nutrient receptors, which detect environmental stimuli. Downstream of such receptors, receptor-specific signal transduction pathways lead to the activation of dynamic TFs, including EGR1, EGR2, NF-κB, and STATs, to mediate context-specific gene expression reprogramming (11, 15, 28, 32, 33). For example, lipopolysaccharide (LPS) stimulation of DCs leads to a signaling cascade downstream of Toll-like receptor 4 (TLR4) that results in NF-κB activation and translocation into the nucleus. NF-κB activates the transcription of thousands of LPS-response genes necessary to orchestrate inflammation (22). Similarly, type I IFNs stimulate STAT1 activation through their receptor, IFNAR. IFNAR activation leads to the activation of interferon signaling genes (ISGs) that include antiviral response genes (34). The ability of these coordinated networks of transcription factors to drive programs of gene expression is intimately linked to the accessibility to regulatory regions such as enhancers and promoters, which is determined by the chromatin landscape.

Integration of context-specific gene expression into epigenetic memory is necessary for DCs to communicate context to other cells once they have migrated away from the site of initial stimulation. The extent to which dynamic changes occurring in the chromatin landscape following stimulation remain stable in rapidly responding, short-lived immune cells such as DCs is not well-understood. While activating TF networks are relatively well-studied in DCs, less is known about the impact of chromatin modifying factors on DC function. Here, we discuss epigenetic mechanisms that have been implicated in the regulation of DC biology, with emphasis on function over differentiation.

## Epigenetic Modifications

DNA methylation, histone modifications and chromatin accessibility are the most well-studied mechanisms that regulate gene expression (35–37). Implicated regulatory proteins are known as “readers,” “writers,” or “erasers” that detect, deposit or remove histone modifications, respectively. Histone modifications and associated regulatory proteins are continually being identified and our understanding of the mechanisms by which they regulate gene expression are continually refined [Table 1; (44, 45)]. ATAC-seq, (Assay for Transposase Accessible Chromatin coupled to sequencing) gives an overall picture of chromatin accessibility irrespective of specific modifications and can be performed on few cells (46). Recently, a fairly comprehensive atlas of chromatin accessibility of 86 immune cells, including 5 DC subsets, was reported (47). These data provide key insights to the overall differences in the chromatin landscape among immune cells and serve as a foundation to more extensively study the mechanisms underlying the diverse and dynamic chromatin architecture in immune cells.

**Table 1:** Enzymes mediating epigenetic modifications.

**Enzyme family**	**Examples**	Catalyzed residue(s)^*^	**Transcriptional response**
DNA Methyltransferase (DNMT)	DNMT1	Cytosine	Repression (Activation)
	DNMT3a		
	DNMT3b		
DNA Demethylase	TET1-3	5-methylcytosine (5mC)^**^	Activation
Histone Deacetylase (HDAC)	HDAC1-11	K residues, specificity unknown	Repression
	SIRT1	H1K26; H3K9, K14, K56; H4K16	
	SIRT2	H3K56; H4K16	
	SIRT3	H4K16	
	SIRT4-5	None	
	SIRT6	H3K9, K56	
	SIRT7	H3K18	
Histone Acetyltransferase (HAT)	HAT1	H2AK5; H4K5, H4K12	Activation
	p300	H2AK5; H2BK5, K12, K15, K20; H3K9, K14, K18, K23, K27; H4K5	
	CBP	H2AK5; H2BK12, K15; H3K18, K23, K27	
	hGCN5	H3K9, K14, K18, K23	
	Tip60	H2AK5, H3K14, H4K5	
	PCAF	H3K14	
	SRC-1	H3K9, K14	
	OGA	H3K14	
	CLOCK	H3K14	
	hMOF	H4K16	
	ATF2	H2BK5, K12, K15; H4K5	
Histone Methyltransferase (HMT)	KMT1A-B	H3K9	Repression
	KMT1C	H3K9, H3K27, H3K56	
	KMT1D	H3K9, H3K27	
	KMT1E-F	H3K9	
	KMT2A-G	H3K4	Activation
	KMT2H	H3K4, H3K36	
	KMT3A	H3K36	
	KMT3B	H3K36, H4K20	
	KMT3C	H3K4, H3K36	
	KMT4	H3K79	
	KMT5A-C	H4K20	Repression
	KMT6	H3K9, H3K27	
	KMT7	H3K4	Activation
	PRMT5	H3R8	Repression
	PRMT6	H3R2	
	CARM1	H3R2, R17, R26	Activation
	PRMT1	H4R3	
Lysine Demethylase (KDM)	KDM1A	H3K4, H3K9	Repression
	KDM1B	H3K4	
	KDM2A	H3K36	
	KDM2B	H3K36, H3K4	
	KDM3A-B	H3K9	Activation
	JMJD1C	H3K9	
	KDM4A	H3K9, H3K36, H1.4K26	Activation/Repression
	KDM4B	H3K9, H3K36, H1.4K26	
	KDM4C	H3K9, H3K36, H1.4K26	
	KDM4D	H3K9	Activation
	KDM5A-D	H3K4	Repression
	KDM6A	H3K27	Activation
	KDM6B	H3K27	
	KDM7A	H3K9, H3K27	
	KDM8	H3K36	Repression
	PHF8	H3K9	Activation
	PHF2	H3K9	
	NO66	H3K4, H3K36	Repression
E3 ligase activity	RING1A	H2AK119ub1	Repression
	RING1B		

* *Lysine (K), arginine (R)*.

** *TET catalyzes 5mC to 5-hydroxymethylcytosine (5hmC), which will be repaired by thymine-DNA glycosylase (TDG) to yield non-methylated cytosine. Enzyme families reviewed in Jones (36), Di Croce and Helin (38), Seto and Yoshida (39), Keating and El-Osta (40), Kampranis and Tsichlis (41), D'Oto et al. (42), and Kohli et al. (43)*.

### DNA Methylation

DNA methylation of cytosine residues (5-methylcytosine; 5mC) occurs in the context of CpG dinucleotides and is mediated by the family of DNA methyltransferases (DNMTs) (36, 48, 49). Sites of DNA methylation are relatively stable, and are propagated through DNA replication during cell division. *De novo* methylation is mediated by DNMT3A/B whereas the reliable transmission of DNA methylation from a mother cell to a daughter cell depends on DNMT1 linked to the replication machinery (50). CpG-rich regions, termed CpG islands, are typically unmethylated but can be aberrantly methylated in cancer and during aging (51, 52). The relationship between CpG methylation and gene regulation is complex. Methylation in promoter regions leads to silencing, whereas methylation in the gene body may facilitate gene expression (36). Proteins containing methyl-CpG binding domains (MDB), C2H2 zinc fingers, or SET-RING finger-associated (SRA) domains that recognize methylated DNA generally promote gene repression, however can also mediate gene activation (53, 54).

Loss of 5mC can occur passively through cell division (where methylation is not copied) or can be actively mediated in a replication-independent manner by Ten eleven translocation (TET) hydroxylases (48). TET hydroxylases catalyze the oxidation of 5mC to 5-hydroxymethylcytosine (5hmC) in an Fe^2+^- and α-ketoglutarate-dependent manner (55). 5hmC can be iteratively oxidized by TET enzymes to other oxidized cytosines that are recognized and excised by thymine DNA glycosylase and replaced with an unmodified cytosine by base-excision repair. 5hmC is found in promoter gene bodies of actively transcribed genes, suggesting that it may have functions other than mediating DNA demethylation (48, 56, 57).The importance of TET enzymes and 5hmC for differentiation of lymphoid and myeloid cells is well-established; however, roles for DNA methylation and 5hmC in regulating immune cell function have been addressed predominantly in lymphoid cells (48).

Consistent with the role of DNA methylation in regulating cellular differentiation programs, several *in vitro* studies have found that DNA methylation is significantly remodeled during DC differentiation. Cultured monocytes can differentiate to multiple lineages, depending on the cytokine cocktail provided. GM-CSF alone, or in combination with IL-4, will stimulate DC differentiation, while a GM-CSF, IL-4 and prostaglandin E2 (PGE2) cocktail will promote differentiation to monocyte-derived suppressor cells (MDSCs) (58–61). IL-4 signaling promotes DC differentiation by activating STAT6. STAT6 promotes the expression of DC-specific genes by recruiting TET2, which results in demethylation and increased DC-specific gene expression (62). PGE2 promotes MDSC differentiation by activating DNMT3A, which methylates and suppresses proinflammatory genes, thus supporting an immunosuppressive phenotype (63). The DNA methylome may serve to prime lineage-specific proinflammatory genes for rapid transcriptional activation upon encounter of appropriate stimuli (64). Though the DNA methylome is thought to be remarkably stable, at least one study has demonstrated that bacterial infection of human DCs leads to rapid DNA demethylation in the absence of cell division (65). In this case, loss of DNA methylation occurred most frequently at enhancers and was associated with the recruitment of dynamic TFs. Increased 5hmC levels were also detected, strongly implicating TET proteins in this process. Thus, surveying the genome-wide DNA methylation profile of DCs can reveal cellular adaptation patterns to extrinsic stimuli, particularly in the context of DC development and differentiation, and in the context of infection. Annotating DNA methylation to gene bodies, promoter regions or other regulatory regions may clarify the contribution of DNA methylation to gene expression programs in DCs. Furthermore, because DCs do not divide following stimulation, monitoring both 5mC and 5hmC may also shed light on dynamic changes in epigenetic control of key genes that regulate inflammatory function of DCs.

### Histone Modification

The enzymatic addition or removal of chemical groups to histone tails regulates chromatin structure and therefore the location and activity of regulatory factors that control transcription. The most widely studied histone modifications are acetylation, methylation and ubiquitylation [Table 1; (38–43)]. The histone code refers to the combination of these modifications that collectively determines the outcome of gene expression (37). In general, transcriptional activation is associated with acetylation of lysine residues of histones, which promotes a more relaxed chromatin structure. Acetylation is mediated by histone acetyltransferases (HATs) and removed by histone deacetylases (HDACs). Histone methylation, on the other hand, is associated with both transcriptional activation and transcriptional repression. There are many described histone methyltransferases (HMTs) and lysine demethylases (KDM) that target a range of lysine and arginine residues (Table 1). Ubiquitination has been mostly studied in the context of the E3 ligase Really Interesting New Gene (RING) proteins that are associated with polycomb repressive complex 1 (PRC1) and deposit ubiquitin on H2A.

Profiling a set of well-studied histone marks can give an overall picture of the activity of a given gene or regulatory region. H3K36me3, H3K27Ac, and H3K4me3 are commonly enriched at active genes, whereas H3K27me3 and H3K9me3 are enriched at silenced genes. H3K4me1 is often found at enhancers while H3K4me3 is enriched at active promoters (66). An enhancer is considered “poised” if it carries H3K4me1 alone or in combination with H3K27me3, and is considered active if H3K4me1 is in combination with H3K27ac (67–70). The genome-wide histone modification profile helps determine cellular identity in part by instructing binding events at specific chromosomal loci; histone modifications can alter the accessibility of transcriptional machinery at underlying genes, or can serve as beacons to recruit chromatin remodelers to either detect, deposit, or remove these histone marks (71). Any irregularities in this system can thus threaten cellular identity, potentially initiating disease (72, 73). Further, several studies have argued these irregularities to be the result of an emerging player in chromatin dynamics: altered cellular metabolism (40).

## Intersection Between Immunometabolism and Epigenetics

**Figure 1:**
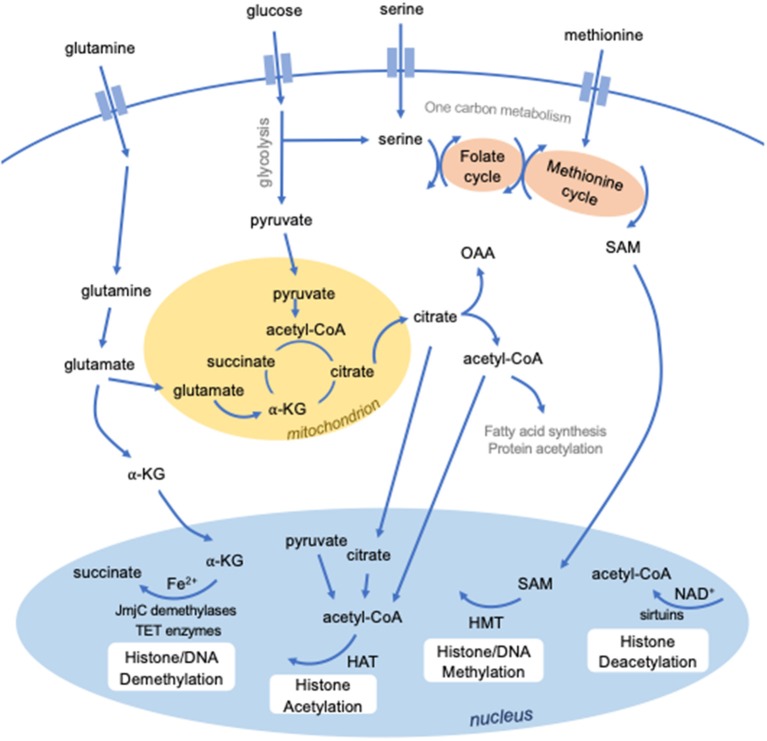
Intersection between metabolism and epigenetics. Several metabolites are required to mediate epigenetic modifications. S-adenosylmethionine (SAM), derived from methionine and one-carbon metabolism, is used for methylation by histone methyltransferases (HMTs). Certain classes of enzymes responsible for histone (JmjC domain-containing demethylases) or DNA (TET enzymes) methylation are dependent on Fe2^+^ and alpha-ketoglutarate (a-KG). Histone acetylation by histone acetyltransferases requires the metabolic intermediate acetyl-CoA, which can be derived from several sources, including pyruvate, citrate, and cytosolic acetyl-CoA. Histone deacetylation by a class of histone deacetylases known as sirtuins require NAD^+^.

The enzymes that modify histones and DNA require specific metabolites as substrates and cofactors (Figure 1). Epigenetic modifications are therefore dependent on the availability of these metabolites and the metabolic pathways used by the cell. In turn, metabolic programming is controlled by epigenetics. Therefore, epigenetic and metabolic control of cellular function intersect at many levels.

Cellular metabolism is central to the regulation, function and activation of immune cells, including DCs. Glycolysis is a major metabolic pathway that rapidly generates energy by breaking down glucose into pyruvate in the cytosol. Pyruvate can enter the mitochondria and feed into the tricarboxylic acid (TCA) cycle, which produces reducing agents that donate electrons to the electron transport chain. This powers highly efficient energy production through a process called oxidative phosphorylation. Importantly, glycolysis and the TCA cycle generate intermediates that feed into numerous other metabolic pathways. Upon TLR stimulation, DCs shift their metabolic activity to glycolysis, and inhibiting this shift impairs DC activation (74, 75). The increase in glycolytic activity increases pyruvate, and subsequently citrate levels, to fuel fatty acid synthesis required to support the rapid membrane expansion that accompanies DC activation (75). Despite their similarity, cDC1s and cDC2s have recently been described to possess distinct metabolic phenotypes that are essential for their differing priming functions, with cDC1s displaying much greater oxidative metabolism (76). The epigenetic factor, polycomb group factor 6 (PCGF6), which has been found to maintain DC quiescence and limit DC activation by negatively regulating H3K4me3 levels, also impairs early glycolytic activity, as measured by extracellular acidification rate (77). Whether, PCGF6 partly limits DC activation by regulating chromatin accessibility of genes important for certain metabolic pathways is unknown.

How cellular metabolism and differing metabolic states affect the DC epigenome requires further investigation; however, several studies of conserved pathways in other innate immune cell types provide insight. Methylation requires methyl groups provided by S-adenosyl methionine (SAM), which is generated from ATP and methionine. Limiting SAM levels can weaken the innate immune response in *Caenorhabditis elegans* against the bacterial pathogen *Pseudomonas aeruginosa* by reducing the levels of H3K4me3 at protective bacterial response genes (78). Furthermore, demethylation requires Fe^2+^ and α-ketoglutarate (α-KG), as cofactor and cosubstrate, respectively, for JmjC domain-containing histone demethylases as well as TET enzymes. In macrophages, the α-KG/succinate ratio regulates the activity of the H3K27 demethylase JMJD3, with higher α-KG promoting JMJD3 activity at genes associated with M2 activation (79). In this instance, IL-4, which induces M2 polarization, stimulates glutaminolysis to generate α-KG to both promote JMJD3 activity as well as to suppress the NF-κB pathway by activating another α-KG-dependent enzyme, prolyl hydroxylase (79). Like succinate, several other metabolites can compete with α-KG to inhibit α-KG-dependent enzymes, including fumarate and 2-hydroxyglutarate (80, 81). Adjusting the balance of these substrates allows innate immune cells to fine-tune and modulate demethylase activity in response to external stimuli, consequently regulating their gene expression programs.

The availability of acetyl-CoA, an intermediate in several anabolic and catabolic pathways, is known to influence histone acetyltransferase activity (82). Several metabolites have also been described to activate or inhibit histone deacetylase activity (83). Importantly, a class of histone deacetylases known as sirtuins (SIRT) are dependent on the oxidizing agent NAD^+^ (84). During sepsis, SIRT1 and SIRT6 are responsible for a switch in metabolic phenotype from glycolysis during early acute inflammation to fatty acid oxidation in the late immunosuppressive phase (85). SIRT1 and endogenous NAD^+^ levels increase simultaneously during endotoxin tolerance, promoting SIRT1 binding and deacetylation at the TNFα promoter, therefore repressing *TNFA* transcription (86). These findings were demonstrated in THP-1 human promonocyte cells, murine splenocytes, and whole blood leukocytes of human sepsis patients. In contrast, short-chain fatty acids produced by the gut microbiota inhibit histone deacetylase activity (87). The most potent of these short-chain fatty acids is butyrate, which contributes to immune tolerance to commensal bacteria by inhibiting proinflammatory functions of intestinal macrophages (88). Clearly, the functions of innate immune cells are regulated by the exquisite interconnection between epigenetic and metabolic reprogramming. Further studies are required to identify the importance of metabolic-epigenetic interactions for DC function.

## Epigenetic Regulation of DC Functions

Expanding evidence suggests that epigenetic modifications contribute significantly to the regulation of DC function. Epigenetic mechanisms are implicated in the maintenance of the steady-state, responses to activating stimuli, trained immunity, and tolerance (Table 2). Furthermore, metabolism, nutrition, environment, and aging also impact DC function by influencing the epigenetic landscape. Ultimately, these mechanisms are important to understand as they impact immune responses to infections and cancers and contribute to inflammatory diseases such as autoimmunity and asthma.

**Table 2:** Epigenetic factors that influence DC activity.

	**Enzyme**	**Function**	**Known target genes in DCs**	**Notes**	**References**
Promotes DC activation	KDM6B (JMJD3)	H3K27 demethylase	*Cd80, Cd86, CD103*		(89)
	WDR5	H3K4 methyltransferase	*IFNA, IFNB*		(90)
	KDM4D (JMJD2D)	H3K9 demethylase	*Il12, Il23*	Recruited by Trabid	(91)
	NuRD complex (HDAC1, HDAC2)	Histone deacetylation complex	*Tnfrsf9, Cd40, Cd80, Cd86, Cd68, Slc11a, Ciita. H2-Aa*	Recruited by Mbd2	(92)
	HDAC11	Histone deacetylase	*IL10*		(93)
Promotes DC steady-state	PCGF6	Transcriptional repressor	*Ciita, H2-Ab1, Il12a, Il12b*	Forms complex with KDM5C	(77)
	KDM5B	H3K4 demethylase	*Ifnb, Il6, Tnfa*	Upregulated by RSV	(94)
	HDAC2	Histone deacetylase	*Il6*	Recruited by Tet2	(95)
	G9a	H3K9 methyltransferase	*Ifna, Ifnb*		(96)

### Active Maintenance of DC Homeostasis

Maintaining DC homeostasis requires balancing of the mechanisms that repress activation and those that promote proinflammatory functions. Clues from the study of TFs suggest that active restraint of DC activation is regulated at the level of transcription. NF-κB, which is recognized to have pioneer factor activity, has been widely shown to induce inflammatory gene expression programs in part by promoting chromatin remodeling (97, 98). At steady state, NF-κB restrains DC activation and prevents DCs from inducing self-reactive cytotoxic T cell responses (99). Deficiency of the p50 subunit of NF-κB in DCs leads to the spontaneous induction of diabetes in a mouse model (99). However, NF-κB activity is also well-known to drive DC activation. One study, using genome footprinting and chromatin immunoprecipitation (ChIP), revealed that the promoter of the MHC class II transactivator *CIITA* is occupied by NF-κB (p65) at steady-state but not in activated DCs, suggesting that NF-κB relocates when DCs become activated (100). Whether the chromatin landscape dictates NF-κB binding in steady-state vs. activated state remains to be determined.

Interestingly, lineage-specific factors that contribute to the differentiation of DCs have also been described to be downregulated in response to maturation signals (101–103). For example, expression of ZBTB46, a zinc-finger DNA-binding TF, is restricted to cDCs. Downregulation of ZBTB46 accompanies TLR-stimulation and is necessary to permit activation (101, 104). Once committed, the lineage of DCs is highly stable (4, 28), therefore it is possible that sustained expression of lineage-specifying factors may serve to restrain the full maturation of DCs until the appropriate activating signals are received.

**Figure 2:**
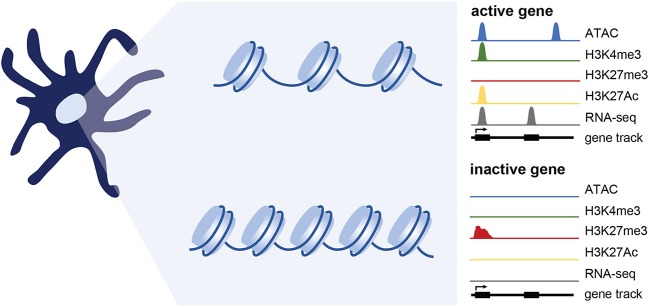
Epigenetic changes associated with gene expression. Simplified representation of data profiles of ATAC-seq, ChIP-seq, and RNA-seq showing an active or inactive gene. Active genes are accessible (measured using ATAC-seq) and bear chromatin modifications associated with transcriptional activation such as H3K4me3 and H3K27Ac. Genes that are inactive are maintained in a repressed, less accessible state and are marked by histone modifications such as H3K27me3. While not extensively tested in DCs, genes poised for expression likely maintain accessibility, and may have a mix of activating and repressive marks.

PCGF6 is a member of Polycomb Repressive Complex 1 (PRC1.6). PRC1 complexes are well-known for catalyzing the monoubiquitylation of histone H2A by a RING E3 ligase (105, 106). H2AK119ub1 leads to chromatin compaction and gene silencing. PCGF6 participates in non-canonical complexes including ones containing E2F6, which promote gene silencing by promoting H3K9 trimethylation, and others that contain KDM5C/D lysine demethylases that remove activating methyl marks at H3K4 (77, 107–112). PCGF6 and KDM5C were both found to be necessary for maintenance of the steady state (77). Following PRR stimulation, PCGF6 is rapidly downregulated, enabling DC activation. PCGF6 regulates the chromatin landscape in DCs and more specifically the levels of H3K4me3 at genes important for DC activation. Though few, these studies suggest that optimal maintenance of the steady state of DCs requires active repression of inflammation-sensitive gene loci through epigenetic silencing at steady state (Figure 2). Rapid relief of transcriptional and epigenetic restraints in response to stimulation is required for massive transcriptional reprogramming that supports DC activation and function.

### Epigenetic Mechanisms Underpin DC Activation and Function

The chromatin landscape at steady state likely dictates the early responses to activating stimuli by regulating accessibility of genes important for DC activation. Following the transcriptional reprogramming that accompanies DC activation, epigenetic reinforcement of gene expression becomes essential to ensure that DCs migrating to lymph nodes retain gene expression profiles to appropriately initiate T cell responses. To activate T cells, DCs must provide at least three signals: antigen presentation (signal 1), co-stimulation (signal 2), and lineage-specifying cytokine production (signal 3). The expression of proteins that constitute these signals are regulated transcriptionally, and increasing evidence suggests they are also regulated epigenetically. In steady-state splenic DCs, the expression of costimulatory molecules *Cd80* and *Cd86* is repressed by H3K27me3, which is relieved by the H3K27 demethylase KDM6B (JMJD3) during LPS stimulation (89). Furthermore, the repressive mark H3K9me3 was found to be enriched at the promoters of proinflammatory cytokines *ll12a, Il12b*, and *Il23* in steady-state BMDCs. Upregulation of these cytokines in LPS-activated BMDCs is largely governed by the recruitment of Trabid, a deubiquitinase that stabilizes the H3K9 demethylase KDM4D (JMJD2D) (91). Nucleosome Remodeling Deacetylase complex (NuRD) also reinforces DC activation by suppressing antigen uptake and processing (*Cd68, Slc11a*) and stimulating antigen presentation (*Ciita, H2-Aa*) (92). This occurs by stabilizing antigen-loaded MHC and by upregulation of specific costimulatory molecules and cytokines. Though these studies suggest that a dynamic epigenome is important for proper DC function, a comprehensive study focused on early and late-stage changes in the chromatin landscape following stimulation and the importance for DC function has not been reported.

Immune mediators in the inflammatory microenvironment such as cytokines, chemokines, and lipids, can temper DC responses to activating stimuli. IL-10 has long been known to potently downregulate IL-12 production (113). HDAC11 represses *IL10* and in doing so, promotes the activation and IL-12 production of primary human DCs, which is required for efficient CD4^+^ T cell differentiation (93). STAT6, a downstream effector of IL-4 signaling, also antagonizes histone acetylation at the *Il10* promoter following LPS stimulation (114). Lipid mediators, such as prostaglandins, can also be sculptors of the epigenome in DCs. Prostaglandin I2 suppresses H3K4me3 enrichment at the *TNFA* promoter by inhibiting components of a methyltransferase complex, MLL and WDR5, from translocating into the nucleus (115). A further study by the same group found that antagonism of the cysteinyl leukotriene receptor promotes an anti-inflammatory phenotype in human moDCs by enhancing H3 acetylation at the *IL10* promoter (116). Inhibiting chromatin remodelers could be an effective therapeutic avenue for inflammatory conditions, in particular those driven by TNFα or controlled by IL-10. Together these studies demonstrate that epigenetic mechanisms significantly contribute to the activation of DCs, and importantly, that factors in the inflammatory environment that modify the epigenome may have lasting effects on DC responsiveness.

### Trained Immunity and Tolerance

The response of myeloid cells can be influenced by previous exposure to inflammatory stimuli. Exposure of DCs and macrophages to low levels of endotoxin induces tolerance which decreases their sensitivity to subsequent stimuli. Exposure of cells to stimuli that increases subsequent responsiveness is termed “trained immunity” and is most commonly noted in monocytes; whether trained immunity is transferred to monocyte-derived DCs upon differentiation is not known. There is increasing evidence that epigenetic and metabolic programming underlies tolerance and training of myeloid cells (98, 117).

Tolerance in myeloid cells, including DCs, is a refractory period following proinflammatory stimulation whereby the immune system is non-responsive to subsequent threats. During sepsis, for example, tolerance serves as a protective mechanism to prevent endotoxin shock in the host. In this state, monocytes, DCs, and macrophages adopt a chromatin landscape that predominantly favors immune suppression (29, 31). This is in part accomplished by the upregulation of suppressive factors such as IL-10, PD-L1, IDO, and TGFβ, along with concomitant silencing of IL-12 and other proinflammatory mediators. These changes in gene expression are accompanied by changes in H3K27me3, H3K27Ac, and H3K4me3 enrichment (29, 31, 118). HDAC2 activity at the *Il6* promoter during late-stage inflammation can lead to *Il6* downregulation and a subsequent return to homeostasis (95).

Training of monocytes by β-glucan stimulation leads to epigenetic and metabolic alterations that prime proinflammatory genes for enhanced expression in response to further stimulation (117). Bacillus Calmette-Guérin (BCG) exposure also trains monocytes to enhance their responses against *Mycobacterium tuberculosis* infection (119, 120). Training can occur at the level of hematopoietic stem cells, leading to unique epigenetic and metabolic signatures in macrophages arising from BCG-trained monocytes (120). BCG and β-glucan training is dependent on glycolysis induced through key metabolic regulators mTOR and HIF-1α (119, 121). Innate immune memory may also occur in microglia, myeloid cells in the brain, affecting neuropathology in murine models of stroke and Alzheimer's. HIF-1α levels are similarly increased in the trained microglia suggesting metabolic reprogramming may underlie training (122). A transcriptomics and metabolomics approach uncovered that glycolysis, glutaminolysis and cholesterol synthesis are essential metabolic pathways for inducing the trained phenotype in monocytes (123). Fumarate accumulation resulting from increased glutaminolysis leads to inhibition of histone demethylases and an increase in H3K4me3 marks at the promoters of proinflammatory cytokines. In addition, mevalonate, a metabolite from the cholesterol synthesis pathway, induces trained immunity by autocrine signaling through IGF1 receptor and subsequent mTOR activation (124). Collectively, these studies suggest that cells of the myeloid lineage undergo epigenetic and metabolic reprogramming in response to environmental stimuli that alters subsequent responses to stimuli. The extent to which environmental stimuli alters metabolic and epigenetic programming of DCs and alters their subsequent responses remains to be studied in detail.

### Viral Infection

The study of antiviral immunity has provided key insights into the contribution of epigenetic mechanisms to DC activation. For instance, interferon production by human DCs can be activated or suppressed by functionally dichotomous chromatin remodelers; the H3K4-specific methyltransferase WDR5 stimulates antiviral immunity via H3K4 trimethylation at the *IFNA* and *IFNB* promoters (90), while H3K9me2 enrichment by the histone-lysine N-methyltransferase G9a at *IFNA* and *IFNB* promoters instead correlates with a decreased DC-driven antiviral response (96). Although a practical system to ensure appropriate interferon expression, certain pathogens have evolved strategies to hijack these endogenous epigenetic pathways and skew the epigenetic signature in their favor. Respiratory syncytial virus (RSV) infection can be cleared by a T_H_1 cytokine profile, but RSV-infected patients often mount a T_H_2 cytokine response non-conducive to efficient RSV clearance. One group found aberrant T_H_2 responses to be driven by an RSV-mediated upregulation of endogenous H3K4 demethylase KDM5B in several DC types, a transcriptional repressor of T_H_1-associated cytokines important for RSV clearance (94). Furthermore, during viral infection in mice, TET2 is recruited by CXXC5 to the *Irf7* promoter to induce *Irf7* hypomethylation and expression in pDCs, resulting in the onset of an antiviral response (125). Given the role of TET2 in stabilizing HDAC2 at the *Il6* promoter (as described earlier), TET2 drives dichotomous DC functions; while TET2 can recruit HDAC2 to help repress *Il6* and resolve IL-6-driven inflammation, it can also initiate an inflammatory antiviral response by hypomethylating and upregulating *Irf7* expression. Advances in both the understanding of the biochemical function of 5hmC and TET enzymes in DCs are necessary to fully appreciate the role of dynamic changes in DNA methylation for regulating gene expression during infection.

## Environmental Factors

A hallmark of the epigenome is its proclivity to undergo extensive remodeling in response to environmental stimuli. Though understudied, accumulating evidence demonstrates that extrinsic factors (in addition to microbes and inflammatory mediators), such as nutrients, chemicals and even aging, can manipulate DC function by altering the epigenetic landscape [Table 3; (139)].

**Table 3:** Environmental factors that shape the epigenome in DCs.

**Extrinsic agent**	**Effect on DC function**	**References**
Aging	Increase in global DNA hypomethylation	(126)
	Upregulation of *TNFA, IL1A, IL17RC, TLR2, Il23p19*	(127–132)
**Chemicals**		
Phthalates	Enhance T_H_2 allergic responses	(133)
	Downregulate *IRF7*	(134)
**Nutrition**		
Zinc deficiency	Induces *Il6* promoter demethylation	(135)
Vitamin C	Increases NF-κB activation, IL-12p70 secretion	(136)
	Regulates TET-mediated DNA demethylation (ES cells, lymphomas)	(137, 138)
**Lipid Mediator**		
Prostaglandin I2	Reduces H3K4me3 enrichment at *TNFA* promoter	(115)
Cysteinyl leukotrienes	Reduces H3 acetylation at *IL10* promoter	(116)

### Chemicals and Nutrients

Phthalates, endocrine-disrupting chemicals ubiquitous in the plastic industry, have been shown to possess adjuvant-like properties that enhance T_H_2 allergic responses (133). Phthalates were found to downregulate *IRF7* expression in human pDCs by inhibiting H3K4-specific methyltransferase WDR5 translocation into the nucleus (134). Nutrients from the diet are also known to affect immune cell function through epigenetic regulation. For example, recent estimates suggest a notable zinc deficiency in 65% of the senior population (>65 years old) (140). Zinc deficiency can inappropriately enhance inflammatory responses (141); zinc deficiency was found to correlate with *Il6* promoter demethylation in THP-1 cells, which led to increased IL-6 production and inflammation (135). Several studies have also established vitamin C as a modulator of DNA demethylation (137, 138). Vitamin C can directly regulate TET-mediated DNA demethylase activity in lymphoma and ES cells. Since vitamin C treatment has been shown to increase NF-κB activation and enhance IL-12p70 secretion by BMDCs (136), vitamin C may promote inflammation by demethylation of genes important for DC activation. As we continue to better understand the mechanisms by which nutrition and metabolism regulate cellular physiology, more links are likely to become apparent between these small molecules and epigenetics.

### Aging

Immune aging or “inflammaging” refers to the observed increase in proinflammatory cytokine expression, such as TNFα, by aged innate cells in the absence of acute infection or stimulation (142). Transcriptional dysregulation in many cell types, including non-immune cells, has been shown to correlate with stochastic epigenetic modifications incurred with age, a process known as “epigenetic drift” (143). An early study found a positive correlation between age and global DNA hypomethylation (126), with several later studies reporting demethylation and concomitant dysregulation at key proinflammatory genes, including *TNFA* (127, 128), *IL1A* (129), *IL17RC* (130), and *TLR2* (131). Splenic T cells from aged C57BL/6 mice (>22 months old) show elevated levels of IL-17 secretion (144). Accordingly, increased IL-17 production is also observed in many autoimmune diseases (145), therefore epigenetic drift in DCs may underlie increased age-related incidences in autoimmunity. Indeed, the activation marker H3K4me2 is enriched at the *Il23p19* promoter in aged DCs (132), and IL-23 production is known to play a pivotal role in the maintenance and expansion of T_H_17 immune responses (146). Inflammation ultimately has the capacity to influence epigenetic regulation (147) and therefore may impact age-associated epigenetic changes in immune and non-immune cells. The interconnectivity of these processes likely underlies long-term immune functionality and organismal health.

## Inflammatory Diseases

DCs are an important driver of the inflammation associated with autoimmune disease and allergic asthma. In particular, histone demethylases and hydroxylases containing the JmjC domain, including KDM5C, JMJD2D, and JMJD3, appear to play a significant role in DC-mediated pathogenesis. KDM5C is an important regulator of the steady-state and activation of murine DCs (77). TRABID promotes experimental autoimmune encephalitis (EAE) by stabilizing JMJD2D at the *Il12* promoter, enhancing IL-12 production and immunopathology (91). However, JMJD3 inhibition limits EAE pathology and promotes a tolerogenic DC profile characterized by the reduced expression of CD80/86, and reduced secretion of proinflammatory cytokines IL-6, IFN-γ, and TNFα (89). Several diseases have been linked to aberrant DC methylation profiles in DCs. DNA hypermethylation at the *IRF8* promoter has been noted in Ocular Behcet's Disease (148) and Koyanagi-Harada Disease (149). In both cases, pharmacological DNA demethylation suppressed proinflammatory cytokine production by patient-derived DCs *ex vivo*. In contrast, genome-wide DNA demethylation was observed in the pDCs of patients with systemic lupus erythematosus (SLE), resulting in increased *IFNA* expression which could contribute to SLE onset (150).

Epigenetic modifications have also been described in asthma (151). Upon allergen recognition in the lung, lung-resident DCs upregulate chemokine receptor CCR7, allowing for their migration to the mediastinal lymph nodes, where they prime T cells and promote allergic inflammation. Although several lung-resident DC subsets exist (including cDCs and moDCs), *Ccr7* upregulation is relatively cDC-specific (152). H3K27me3 enrichment was found at the *Ccr7* promoter in moDCs, but not cDCs, suggesting that some lineage specific functions of DCs may be epigenetically determined (153). Mouse studies also suggest asthma risk to be an inherited characteristic partially mediated by an altered DC epigenome. Adoptive transfer experiments in mice identified DCs to be the “carrier” of asthmatic susceptibility; DCs transferred from neonates of asthmatic mothers to neonates of non-asthmatic mothers increased asthma susceptibility in the recipients, indicating a functional skew in DCs early in life that promote allergic responses (154). Donor and recipient mice were genetically identical, suggesting the observed functional skew to be epigenetically regulated. Indeed, the DC methylomes of neonates from asthmatic mothers differed significantly from neonates of healthy mothers, and approximately 50% of the differentially methylated genes belong to allergy and asthma pathology networks (155). Thus, allergen exposure early in life results in alternative epigenetic regulation of key genes that contribute to allergic responses. Thus, the extent to which inflammatory genes are epigenetically primed in DCs likely contributes to inflammatory disease incidence and severity.

## Summary and Perspectives

Because DCs are fast-acting and short-lived, the contribution of epigenetic mechanisms to DC responsiveness and function has been overlooked. However, there is growing appreciation of the importance of epigenetic mechanisms in controlling dynamic, and even short-lived, cellular responses. The past decade has seen exciting advancements in our understanding of how the environment impacts immunobiology at the epigenome level. Significant steps have been taken to understand how the chemicals and nutrients in our environment influence the immune system, as well as the mechanisms by which the aging process contributes to age-related inflammation. The development and use of low-input techniques are necessary to expand epigenetic studies to different *in vivo*-derived DC populations (46, 156, 157). Further studies are needed to expand our knowledge of the mechanisms that regulate the epigenome in DCs and the consequences for healthy and pathological inflammation. DC function is highly influenced by the local environment in which it is stimulated. Thus, environmental factors that shape the epigenome of DCs at steady state are likely to have lasting effects on DC function. Insightful discoveries on the effects of local nutrition, metabolite availability, and inflammation on the epigenetic landscape in DCs will further our understanding of the dynamic changes in gene expression that support or interfere with host immunity.

## Author Contributions

GB, MC, HG, and CK contributed by researching and writing manuscript and creating figures and tables.

### Conflict of Interest Statement

The authors declare that the research was conducted in the absence of any commercial or financial relationships that could be construed as a potential conflict of interest.
